# Independent Validation of EarlyR Gene Signature in BIG 1-98: A Randomized, Double-Blind, Phase III Trial Comparing Letrozole and Tamoxifen as Adjuvant Endocrine Therapy for Postmenopausal Women With Hormone Receptor–Positive, Early Breast Cancer

**DOI:** 10.1093/jncics/pkz051

**Published:** 2019-08-16

**Authors:** Steven A Buechler, Kathryn P Gray, Yesim Gökmen-Polar, Scooter Willis, Beat Thürlimann, Rosita Kammler, Giuseppe Viale, Brian Leyland-Jones, Sunil S Badve, Meredith M Regan

**Affiliations:** 1 University of Notre Dame, Notre Dame, IN; 2 Harper Cancer Research Institute, Notre Dame, IN; 3 IBCSG Statistical Center Department of Biostatistics and Computational Biology, Dana Farber Cancer Institute, Boston, MA; 4 Harvard Medical School, Boston, MA; 5 Harvard T.H. Chan School of Public Health, Boston, MA; 6 Department of Pathology and Laboratory Medicine, Indiana University School of Medicine, Indianapolis, IN; 7 Avera Cancer Institute, Department of Molecular and Experimental Medicine, Sioux Falls, SD; 8 International Breast Cancer Study Group Coordinating Center Pathology Office, Bern, Switzerland; 9 Breast Center, Kantonsspital, St. Gallen, Switzerland; 10 Swiss Group for Clinical Cancer Research SAKK, Berne, Switzerland; 11 Indiana University Melvin and Bren Simon Cancer Center, Indianapolis, IN; 12 University of Milan, IEO European Institute of Oncology IRCCS, Milan, Italy

## Abstract

**Background:**

EarlyR gene signature in estrogen receptor–positive (ER+) breast cancer is computed from the expression values of *ESPL1*, *SPAG5*, *MKI67*, *PLK1*, and *PGR*. EarlyR has been validated in multiple cohorts profiled using microarrays. This study sought to verify the prognostic features of EarlyR in a case-cohort sample from BIG 1–98, a randomized clinical trial of ER+ postmenopausal breast cancer patients treated with adjuvant endocrine therapy (letrozole or tamoxifen).

**Methods:**

Expression of EarlyR gene signature was estimated by Illumina cDNA-mediated Annealing, Selection, and Ligation assay of RNA from formalin-fixed, paraffin-embedded primary breast cancer tissues in a case-cohort subset of ER+ women (N = 1174; 216 cases of recurrence within 8 years) from BIG 1–98. EarlyR score and prespecified risk strata (≤25 = low, 26–75 = intermediate, >75 = high) were “blindly” computed. Analysis endpoints included distant recurrence–free interval and breast cancer–free interval at 8 years after randomization. Hazard ratios (HRs) and test statistics were estimated with weighted analysis methods.

**Results:**

The distribution of the EarlyR risk groups was 67% low, 19% intermediate, and 14% high risk in this ER+ cohort. EarlyR was prognostic for distant recurrence–free interval; EarlyR high-risk patients had statistically increased risk of distant recurrence within 8 years (HR = 1.73, 95% confidence interval = 1.14 to 2.64) compared with EarlyR low-risk patients. EarlyR was also prognostic of breast cancer–free interval (HR = 1.74, 95% confidence interval = 1.21 to 2.62).

**Conclusions:**

This study confirmed the prognostic significance of EarlyR using RNA from formalin-fixed, paraffin-embedded tissues from a case-cohort sample of BIG 1–98. EarlyR identifies a set of high-risk patients with relatively poor prognosis who may be considered for additional treatment. Further studies will focus on analyzing the predictive value of EarlyR signature.

Selection of an effective treatment regimen for a primary breast cancer patient must take into account the molecular heterogeneity of the disease. Approximately 80% of primary breast cancers are estrogen receptor–positive (ER+), almost all of which are treated with endocrine therapy. Although prognosis for early-stage ER+ breast cancer patients treated with endocrine therapy alone is considered “good,” at least 20% of patients will suffer a distant recurrence within 10 years. Traditionally, clinical factors such as tumor size, grade, number of positive lymph nodes, and indicators of proliferation such as Ki67 have been used to assess the utility of adjuvant chemotherapy. However, these factors alone fail to define all molecular traits that have a considerable effect on prognosis. There is a considerable clinical need for a molecular assay that can differentiate between the small percentage of ER+ breast cancer patients who have high risk of relapse and may benefit from chemotherapy and the majority who can safely avoid it.

Multi-gene signatures such as Oncotype DX Recurrence Score (1), Mammaprint (2,3), Risk of Recurrence (ROR) (4), Breast Cancer Index (5), and EndoPredict (6) are increasingly being used to guide treatment decisions. Each test has its advantages and limitations.

To address the limitations of the current assays, we developed the EarlyR gene signature based on the expression of *ESPL1*, *SPAG5*, *MKI67*, *PLK1*, and *PGR* [Supplementary Methods, available online; Buechler et al. (7–9)]. EarlyR can be applied as a continuous score (0–100) or as discrete risk strata (low, intermediate, and high risk). EarlyR has been shown (9) to be prognostic of distant recurrence within 8 years of diagnosis in multiple cohorts with gene expression measured by Affymetrix microarrays and in the Illumina-assayed Molecular Taxonomy of Breast Cancer International Consortium (METABRIC) cohort (10). In the ER+ METABRIC cohort (N = 1518), in particular, EarlyR continuous score and risk strata were statistically significantly prognostic of distant recurrence–free interval (DRFI) and breast cancer–free interval (BCFI), up to 8 years post-diagnosis (hazard ratio [HR] of EarlyR-High to EarlyR-Low for DRFI is 2.6 [95% confidence interval [CI] = 2.0 to 3.3] in ER+ overall), and subgroups of lymph node–negative (LN−), lymph node–positive (LN+), and HER2 negative patients (9). Importantly, in all of these subgroups, EarlyR classified at least 65% of patients as low risk and fewer than 20% as intermediate risk. EarlyR has also been shown to predict response to neoadjuvant chemotherapy (11). We showed that EarlyR-High patients who received neoadjuvant chemotherapy and endocrine therapy had lower risk of recurrence than EarlyR-High patients treated only with endocrine therapy. In contrast, EarlyR-Low patients did not benefit from adding chemotherapy to endocrine therapy.

In most previous studies of EarlyR, gene expression was measured from frozen tissues collected from patient cohorts of convenience, limiting its clinical applicability. Additionally, these patients may not have been treated with regimens that constitute current standard of care. Validation of the prognostic significance of EarlyR in a contemporary, well-annotated, randomized, controlled clinical trial is needed. The current study aims to assess the prognostic significance of EarlyR using a cohort of patients from the randomized, controlled BIG 1–98 clinical trial of endocrine therapy for postmenopausal women with hormone receptor–positive early breast cancer (12).

## Patients and Methods

### Study (EarlyR) Cohort

Between March 1998 and May 2003, 8010 postmenopausal women with hormone receptor–positive operable invasive breast cancer were randomly assigned to monotherapy with letrozole or tamoxifen for 5 years, a sequential strategy of letrozole for 2 years followed by tamoxifen for 3 years, or the reverse sequence (ClinicalTrials.gov NCT00004205). At the protocol-specified update 12 years after trial commencement, and with a median follow-up of 8.1 years, 1022 distant recurrences were observed (12). Chemotherapy treatment was at the discretion of individual physicians and patients.

Between 1998 and 2010, the International Breast Cancer Study Group (IBCSG) carried out retrospective tissue collection in accordance with institutional guidelines and national laws. Formalin-fixed, paraffin-embedded (FFPE) primary breast cancer tissue blocks were assessed for availability of invasive tumor material for translational research under the approval of the IBCSG Biological Protocols Working Group. All material processing and assaying was done without knowledge of patients’ treatment assignments or outcomes. Appropriate permissions from the University of Notre Dame and Indiana University institutional review boards were also obtained for use of the samples and data.

A case-cohort sampling design was used to select 1218 FFPE tissue samples (DASL cohort; 257 cases of recurrence) for whole-genome profiling using the DASL protocol on the Illumina HT-12 v4 microarray as follows. All breast cancer recurrence cases (ie, BCFI events) with available RNA were included, and nonrecurrence cases were sampled according to four stratification factors, resulting in 48 strata (classes). There were differences in the proportion of nonrecurrence patients sampled by the four stratification factors: (1) two geographic regions; (2) treatment arms and randomization option, resulting in six groups; (3) lymph node status (positive, negative/unknown); and (4) prior chemotherapy use (no, yes). The sampling fractions were the number of cases selected for DASL analysis divided by the number of BIG 1–98 RNA samples in each of these 48 groups. The weights were the inverses of the sampling fractions.

**Figure 1.**
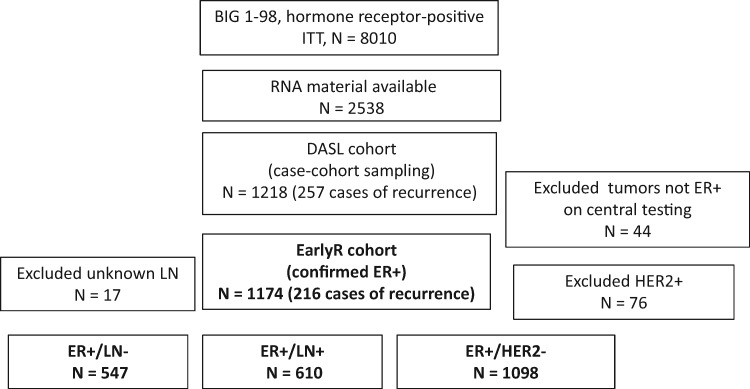
Flow diagram for defining the EarlyR cohort and analytic subgroups. DASL = Illumina’s cDNA-mediated annealing, selection, extension, and ligation; EarlyR = a prognostic risk score defined using the selected five genes (*ESPL1*, *SPAG5*, *MKI67*, *PLK1*, and *PGR*) panel; ER+ = estrogen receptor–positive; ITT = intention to treat; LN+ = lymph node–positive; LN− = lymph node–negative.

The EarlyR cohort (N = 1174; Figure 1) consists of all samples from the DASL cohort (N = 1218) that were centrally confirmed to be ER+ (≥1% ER expression by immunohistochemistry). The prognostic significance of EarlyR was also analyzed in three subcohorts of ER+/LN− (N = 547), ER+/LN+ (N = 610), and ER+/HER2− (N = 1098) patients, where centrally assessed ER (positive is ≥1% immunostained cells), HER2 status (positive if amplified by fluorescent in situ hybridization (FISH) or a few cases that were 3+ by immunohistochemistry without fluorescent in situ hybridization assessment), and lymph node status were used to define the subcohorts.

### Gene Expression Analysis

Gene expression was quantified for samples in the DASL cohort before initiation of this project as follows. Centrally extracted mRNA specimens were profiled using the Illumina Whole-Genome DASL protocol for expression profiling with Illumina HT-12 v4 BeadArray platform for FFPE samples (under the direction of S.W. and B.L.-J.) at Florida Scripps. Gene expression values were cubic spline normalized with no background correction using BeadStudio software (Illumina). The natural logarithms of microarray probe expression values were used in this analysis. Expression values for probes other than the five used to compute EarlyR were not available for this project. Access to these data may be requested through the IBCSG data-sharing process.

### Computation of EarlyR Score and Risk Strata

Expression values for the EarlyR panel genes were obtained from the Illumina DASL assay data for the study cohort using the same Illumina probes used to compute EarlyR in the METABRIC cohort (9), specifically *ESPL1* (ILMN_1742145), *MKI67* (ILMN_1734827), *SPAG5* (ILMN_2141259), *PLK1* (ILMN_1736176), and *PGR* (ILMN_1811014). The EarlyR score (0–100) was computed for each sample as described in the Supplementary Methods (available online). This computation was performed (by SA Buechler) blinded to the patients’ clinical data. The EarlyR risk strata were subsequently defined using predetermined thresholds (9) as follows: EarlyR-Low (EarlyR ≤ 25), EarlyR-Int (EarlyR >25 to ≤ 75), and EarlyR-High (EarlyR >75).

### Study Endpoints

To investigate the prognostic feature of EarlyR with disease outcomes, the analysis endpoints included DRFI, defined as time from randomization to breast cancer recurrence at a distant site, and BCFI, defined as time from randomization to first invasive breast cancer recurrence at a local, regional, or distant site or invasive contralateral breast cancer. Both endpoints were censored at the minimum of last disease follow-up, death or 8 years since diagnosis, in an attempt to focus on the assessment of “early” prognostic features of EarlyR. Eight years was chosen as a threshold based on a prior publication showing that the prognostic utility of current genomic signatures for ER+ breast cancer deteriorates after 8 years (13).

### Statistical Analysis of Disease Outcomes

Sampling weights were computed as described above. Weighted analysis methods (generalized Horvitz-Thompson methods) were used to adjust estimates of recurrence-free interval test statistics to obtain unbiased analyses and to give consistent estimates of prognosis in the full ER+ cohort. In the weighted analyses, contributions to Kaplan-Meier estimators and tests were weighted proportional to the inverses of the sampling fractions; special methods were used to compute variances (14). Weighted Kaplan-Meier estimates of DRFI and BCFI distributions were calculated. From weighted Cox regression, stratified by chemotherapy and treatment assignment, hazard ratios with 95% confidence intervals were estimated, and stratified log-rank trend test statistics with 1 degree of freedom for comparing the three EarlyR risk strata were reported. Similar analyses were used in the exploratory analyses of ER+/LN−, ER+/LN+, and ER+/HER2− subcohorts.

Statistical power was assessed based on the overall ER+ cohort (N = 1174) and the endpoint of BCFI, the endpoint that guided the case-cohort sampling. Due to the case-cohort design, an adjusted standard error estimate for the coefficient of interest (ie, EarlyR three risk strata as a continuous variable) was obtained using the weighed Cox regression model. The standard error was subsequently used to assess statistical power to detect a difference (hazard ratio) comparing two adjacent EarlyR groups based on a trend test. Assuming a two-sided significance level of .05, there was a 90% power to detect a hazard ratio of 1.33 for comparing two adjacent EarlyR risk strata.

Results were presented in accordance with REporting recommendations for tumour MARKer prognostic studies criteria (15). All statistical tests were two-sided. No multiple comparison adjustments were implemented, and *P* values less than .05 in the overall EarlyR cohort were considered statistically significant.

## Results

### Patient Characteristics

Unweighted distributions of clinical features of the EarlyR cohort (N = 1174) were consistent with those of patients enrolled in the BIG 1–98 intention-to-treat population, when considering the case-cohort sampling design (Table 1; Supplementary Table 1, available online). The characteristics of the EarlyR cohort and the three additional analysis subgroups (ER+/LN− [n = 547], ER+/LN+ [n = 610], and ER+/ HER2− [n = 1098]) are summarized in Table 1. In the EarlyR cohort, approximately 30% were assigned to each monotherapy arm and 40% to sequential treatment arms. Chemotherapy was administered to 17% of the LN− and 51% of the LN+ patients. HER2− positivity was 6%. Within 8 years of randomization, the frequencies of DRFI events reported for the ER+, ER+/LN−, ER+/LN+, and ER+/HER2− subgroups were 14%, 10%, 17%, and 13%, respectively, and the frequencies of BCFI events reported were 18%, 15%, 21%, and 17%, respectively, for the EarlyR cohort and the three analysis subgroups.

**Table 1.** Characteristics of the overall EarlyR cohort and analytic subgroups (unweighted distributions)*

Characteristic	ER+ (N = 1174)	ER+/LN− (N = 547)	ER+/LN+ (N = 610)	ER+/HER2− (N = 1098)
Treatment assignment, No. (%)				
Letrozole	333 (28)	163 (30)	164 (27)	301 (27)
Tamoxifen	362 (31)	180 (33)	176 (29)	339 (31)
Sequential	479 (41)	204 (37)	270 (44)	458 (42)
Age, median (IQR), y	61 (56–68)	62 (56–68)	61 (55–68)	62 (56–68)
Mastectomy, No. (%)	417 (36)	154 (28)	260 (43)	383 (35)
Prior chemotherapy, No. (%)	403 (34)	93 (17)	310 (51)	368 (34)
Tumor size >2 cm, No. (%)	465 (40)	164 (30)	296 (49)	426 (39)
Tumor grade, 2 or 3, No. (%)	992 (85)	448 (82)	529 (87)	916 (84)
Lymph node positive, No. (%)	610 (53)	0 (0)	610 (100)	573 (53)
ER positive, No. (%)	1174 (100)	547 (100)	610 (100)	1098 (100)
HER2-, No. (%)	1098 (94)	511 (93)	573 (94)	1098 (100)
Ki-67 LI of immunostained cells, median (IQR), %	13 (7–21)	12 (7–21)	14 (8–20)	12 (7–20)
Recurrences, No. (%)				
8 y breast cancer event	216 (18)	84 (15)	127 (21)	190 (17)
8 y distance recurrence	163 (14)	55 (10)	104 (17)	145 (13)
EarlyR risk group, No. (%)				
EarlyR-Low	792 (67)	383 (70)	396 (65)	756 (69)
EarlyR-Int	161 (14)	61 (11)	99 (16)	141 (13)
EarlyR-High	221 (19)	103 (19)	115 (19)	201 (18)

*EarlyR-High (75 < EarlyR), EarlyR-Int (25 < EarlyR ≤ 75), and EarlyR-Low (EarlyR ≤ 25); IQR = interquartile range.

### Unweighted Distribution of EarlyR

**Figure 2.**
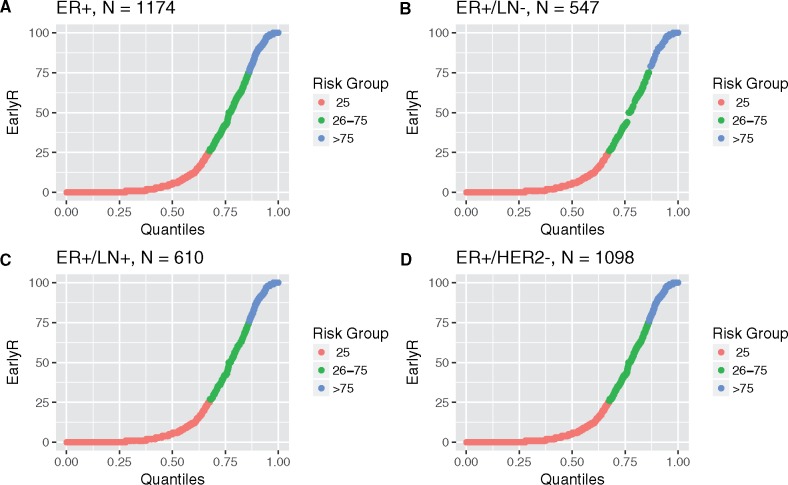
EarlyR continuous score is plotted vs quantiles of the score for analytical cohorts (**A**) ER+, (**B**) ER+/LN−, (**C**) ER+/LN+, (**D**) ER+/HER2− in this study. The three EarlyR risk groups, defined with predetermined thresholds of 25 and 75, are indicated by color. In each cohort, the low-risk group (EarlyR ≤25) contains at least 65% of patients, and the intermediate-risk group (EarlyR >25 to ≤75) contains less than 20% of patients. EarlyR = a prognostic risk score defined using the selected five genes (ESPL1, SPAG5, MKI67, PLK1, and PGR) panel; ER+ = estrogen receptor–positive; LN+ = lymph node–positive; LN− = lymph node–negative.

In the primary EarlyR cohort of all ER+ breast cancer patients (N = 1174), EarlyR score (Figure 2A) classified 67% of patients as low risk (EarlyR-Low), 19% as intermediate risk (EarlyR-Int), and 14% as high risk (EarlyR-High). In the secondary analysis subgroups (ER+/LN− [N = 547], ER+/LN+ [N = 610], and ER+/HER2− [N = 1098]), EarlyR-Low contained 65–70% of patients, EarlyR-High contained 11–16% of patients, and EarlyR-Int contained 18–19% of patients (Figure 2B–D; Table 1). These distributions were computed for the actual subgroups and were not weighted estimates.

### EarlyR and Risk of Distant Recurrence and Breast Cancer Recurrence in the ER+ Group

**Figure 3.**
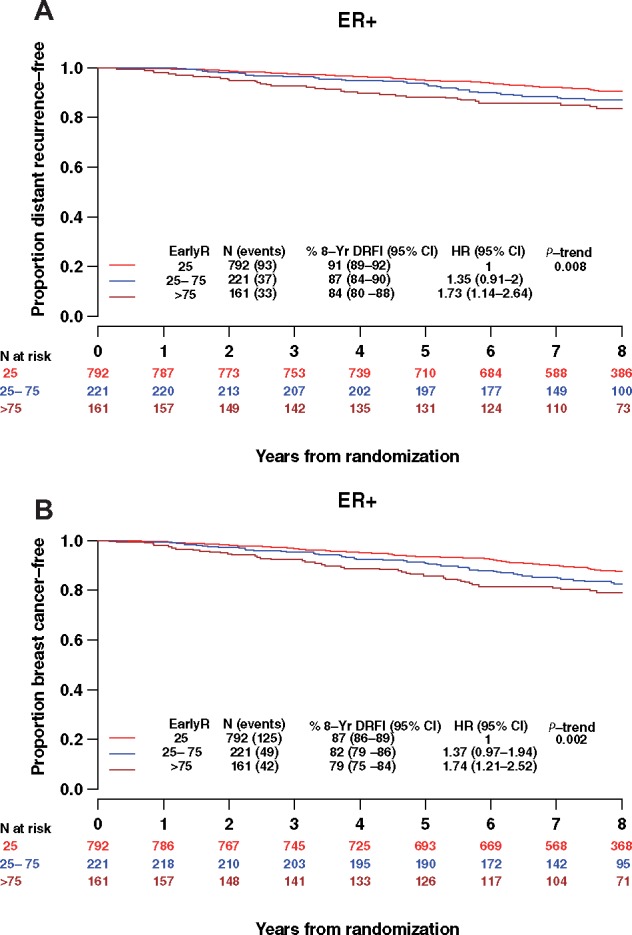
Weighted Kaplan-Meier (KM) estimates of (**A**) distant recurrence–free interval (DRFI) and (**B**) breast cancer–free interval (BCFI) according to EarlyR risk strata in the overall EarlyR estrogen receptor–positive (ER+) cohort. CI = confidence interval; EarlyR = a prognostic risk score defined using the selected five genes (ESPL1, SPAG5, MKI67, PLK1, and PGR) panel; HR = hazard ratio; LN+ = lymph node positive; LN− = lymph negative.

The EarlyR stratification was statistically significantly prognostic of DRFI in the overall ER+ cohort (Figure 3A; *P*_trend_ = .008) within 8 years of randomization; specifically, with weighted HR = 1.73 (95% CI = 1.14 to 2.64) comparing EarlyR-High vs EarlyR-Low or HR = 1.35 (95% CI = 0.91 to 2.0) comparing EarlyR-Int vs EarlyR-Low. The percent free of distant recurrence at 8 years was estimated as 91% (95% CI = 89% to 92%) for EarlyR-Low and 84% (95% CI = 80% to 88%) for EarlyR-High. The continuous EarlyR score was also statistically significantly prognostic. Specifically, for a 10-unit increase of EarlyR risk score, we observed an HR = 1.07 (95% CI = 1.03 to 1.12) for DRFI. In a multivariable model adjusting for age, LN status, tumor size, grade, and Ki-67 labeling index, we observed an HR = 1.03 (95% CI = 0.99 to 1.08) for EarlyR risk score.

Similarly, the EarlyR risk stratification was prognostic of BCFI within 8 years (Figure 3B, *P*_trend_ = .002, HR = 1.74, 95% CI = 1.21 to 2.52 comparing EarlyR-High vs EarlyR-Low; HR = 1.37, 95% CI = 0.97 to 1.94 comparing EarlyR-Int vs EarlyR-Low). The percent estimated free of breast cancer recurrence at 8 years was 87% (95% CI = 86% to 89%) for EarlyR-Low and 79% (95% CI = 75% to 84%) for EarlyR-High. Also, a continuous EarlyR score is prognostic of BCFI with HR = 1.07 (95% CI = 1.03 to 1.11) for a 10-unit increase in the EarlyR risk score.

### EarlyR and Recurrence in Subgroups of ER+ Breast Cancer

**Figure 4.**
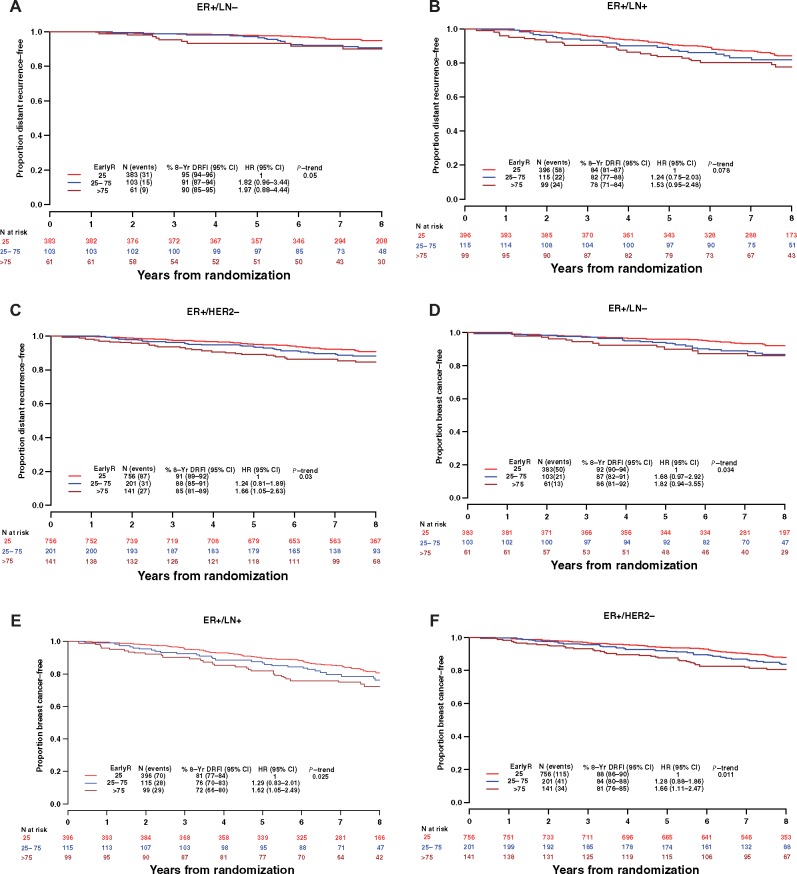
Weighted Kaplan-Meier (KM) estimates of distant recurrence–free interval (DRFI) (**A**–**C**) and breast cancer–free interval (BCFI) (**D**–**F**) according to EarlyR risk strata in the analytic subgroups ER+ (estrogen receptor–positive) lymph node–negative (LN−) (**A**) DRFI, (**D**) BCFI; ER+ lymph node–positive (LN+) (**B**) DRFI, (**E**) BCFI, and ER+ HER2− (**C**) DRFI, (**F**) BCFI. CI = confidence interval; EarlyR = a prognostic risk score defined using the selected five genes (ESPL1, SPAG5, MKI67, PLK1, and PGR) panel; HR = hazard ratio.

As exploratory analyses, we assessed the early (within 8 years) prognostic value of EarlyR in subsets of this ER+ cohort. The EarlyR risk stratification was shown to be prognostic of DRFI in ER+/HER2− (N = 1098, *P*_trend_ = .03; Figure 4C; and similar trends were also observed in ER+/LN− (N = 547, *P*_trend_ = .05; Figure 4A) and ER+/LN+ (N = 610, *P*_trend_ = .08; Figure 4B). EarlyR was also observed to be prognostic of BCFI (Figure 4D–F) in each subgroup. Results were consistent for ER+/LN−/HER2− (N = 511, DRFI *P*_trend_ = .089).

### EarlyR and Recurrence in ER+/LN− Patients Not Treated with Chemotherapy

**Figure 5.**
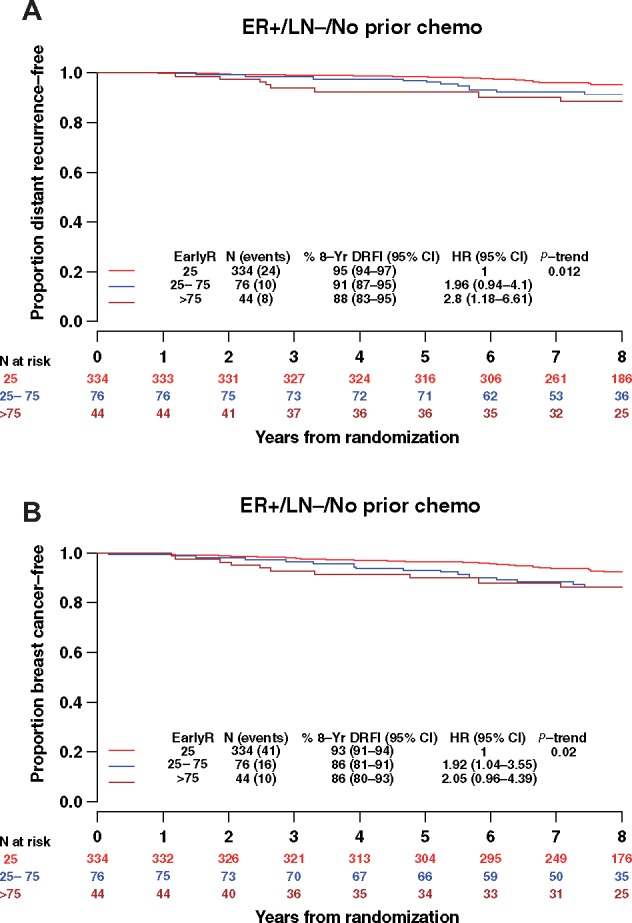
Weighted Kaplan-Meier (KM) estimates of (**A**) distant recurrence–free interval (DRFI) and (**B**) breast cancer–free interval (BCFI) according to EarlyR risk strata in estrogen receptor–positive (ER+) lymph node negative (LN−) patients not receiving chemotherapy. CI = confidence interval; EarlyR = a prognostic risk score defined using the selected five genes (ESPL1, SPAG5, MKI67, PLK1, and PGR) panel; HR = hazard ratio.

The principal application of prognostic signatures such as Onco*type* DX and EarlyR is to discriminate between ER+/LN− breast cancer patients who have sufficiently high risk of relapse to justify treating with chemotherapy and those with good prognosis who are unlikely to benefit from chemotherapy. In the BIG 1–98 trial, chemotherapy treatment was at the discretion of the treating physician. To assess whether physicians left untreated some high-risk patients, we evaluated the prognostic significance of EarlyR in the ER+/LN− patients with no prior chemotherapy treatment (N = 454 of 547). In this subgroup of patients, the estimated 8-year DRFI was 95.4% (95% CI = 94% to 96.8%) in EarlyR-Low (N = 334) and 88.5% (95% CI = 82.7% to 94.6%) in EarlyR-High (N = 44), as shown in Figure 5A. EarlyR was likewise prognostic of BCFI (Figure 5B) in this subgroup. Because most of these patients were HER2− (N = 433), results were consistent in the ER+/LN−/HER2− subgroup.

### Treatment and Recurrence by EarlyR Stratum

In the BIG 1–98 clinical trial, DRFI was statistically significantly improved with letrozole vs tamoxifen monotherapy (12). There were too few events in the EarlyR cohort to properly assess for differential treatment effects by EarlyR stratification. The estimated DRFI as of 8 years was observed to be higher in the letrozole monotherapy arm than that in the tamoxifen monotherapy arm in both EarlyR-Low (Supplementary Figure 1, available online; N = 471, 93.3% vs 88.0%) and EarlyR-High (Supplementary Figure 1, available online; N = 89, 86.1% vs 79.4%).

## Discussion

Clinical trials such as BIG 1-98 have shown the benefit of hormone therapy to treat early-stage ER+ breast cancer in postmenopausal women. Although the majority of early-stage ER+ breast cancer patients remain disease-free when treated with only tamoxifen or an aromatase inhibitor, as many as 20% suffer a distant recurrence within 10 years of diagnosis. Increasingly, multi-gene signatures have proven to be more effective than clinico-pathological traits alone in identifying those ER+ patients who are at a high risk of recurrence and may benefit from cytotoxic chemotherapy in addition to hormone therapy.

The EarlyR genomic signature was shown to be prognostic of DRFI and BCFI in the METABRIC ER+ cohort and of distant metastasis–free survival in multiple other cohorts, independent of clinico-pathological features (9). However, in these prior studies, gene expression was measured from fresh-frozen tissues of samples of convenience, and patient treatments may not have matched current standard of care. It was therefore necessary to validate the gene signature in a randomized clinical trial cohort.

In the current study, we validated the prognostic significance of EarlyR in a case-cohort sample (N = 1174) of BIG 1-98 in which all patients were prescribed endocrine therapy for 5 years. Importantly, mRNA was extracted from FFPE tissues in a single laboratory using standardized protocol and subsequently assayed to measure gene expression.

A strength of this study is the independence of the computation of EarlyR score and strata (Buechler lab), blind to clinical data, from gene expression analysis (Avera Cancer Institute) and statistical analysis (IBCSG Statistical Center). Importantly, the thresholds defining the risk strata were prospectively defined, again blind to clinical data, satisfying the criteria for a prospective study using archived samples defined by Simon et al. (16). The study used a case-cohort design to maximize the use of available samples and a weighted analysis method to give unbiased estimates of prognostic significance of EarlyR in the full ER+ BIG 1-98 study population.

EarlyR was previously shown by concordance index analysis to have comparable prognostic significance to surrogates of the Onco*type* DX Recurrence Score, ROR, and Mammaprint. In this study, prognosis for the EarlyR-Low patients in the overall ER+ postmenopausal breast cancer cohort was excellent, with the estimated 8-year DRFI being 91% (95% CI = 89% to 92%), and in ER+/LN− patients not treated with chemotherapy the estimated 8-year DRFI was 95.4% (95% CI = 94% to 96.8%) and estimated 8-year BCFI was 92.5% (95% CI = 90.8% to 94.3%). This low rate of distant recurrence is comparable with the distant recurrence rates of the low-risk patients identified by Onco*type* DX and PAM50 ROR in the translational protocol of the Arimidex, Tamoxifen, Alone, or in Combination (transATAC) cohort of the ATAC trial (17). In ER+/LN− patients in this study, EarlyR classified 70% as low risk and 11% as high risk. In contrast, in the ER+/LN− patients from transATAC, Onco*type* DX classified 59% as low risk and 10% as high risk, and ROR classified 58% as low risk and 16% as high risk (17). These results build on earlier evidence (9) that EarlyR has comparable or superior prognostic significance to these existing assays while offering a definitive prognosis for more patients.

The risk strata defined for Oncotype DX, ROR, and other tests were selected based on evaluation of candidate thresholds of each test’s continuous score in a training cohort. These different methods naturally lead to some discordance, although in most studies, 50–60% of patients were classified as low risk by each test. In contrast, the risk strata for EarlyR were defined by intrinsic features of the panel genes (Supplementary Methods, available online). In the TAILORx trial, the patients with a recurrence score less than 26 (86%) had an estimated DRFI of approximately 95% after 9 years when treated with hormone therapy alone. The high percentage of TAILORx patients who had good prognosis and did not benefit from chemotherapy is consistent with the definition of a large low-risk stratum by EarlyR.

EarlyR was previously shown to predict the benefit of adding neoadjuvant chemotherapy to hormone therapy (11). The ability of EarlyR to predict adjuvant chemotherapy benefit was not assessed in this study because chemotherapy treatment was not randomized in BIG 1–98. Further studies are needed to determine if EarlyR-High patients are likely to benefit from chemotherapy. As a preliminary step, we showed here that among those ER+/LN− patients not treated with chemotherapy, EarlyR-High patients had poorer prognosis than EarlyR-Low patients.

There are a number of limitations of this study, the principle one being that the gene signature was computed with gene expression data from DASL-whole genome analysis. Although a specific quantitative reverse transcription–polymerase chain Reaction assay has been developed, it was deemed more prudent to use available gene expression data for this study rather than reassaying valuable tissue. Furthermore, due to the case-cohort design of the DASL cohort, hazard ratios and recurrence-free probabilities were estimated using weighted analysis rather than from the whole trial population.

In conclusion, this study confirmed the prognostic significance of the EarlyR signature for postmenopausal patients with ER+ breast cancer assessed using FFPE tissues from a cohort of the BIG 1–98 randomized clinical trial of adjuvant endocrine therapy. Within each analytical subgroup, the EarlyR signature identified a large set (EarlyR-Low, at least 65%) of patients with excellent prognosis and comparably few patients (at most 19%) with intermediate risk. Moreover, in the patients identified as low risk by EarlyR, there was less than 5% risk of distant recurrence at 8 years in the ER+/LN− patients not treated with chemotherapy. Additional studies are needed to determine whether patients identified as high risk by EarlyR are likely to benefit from chemotherapy.

## Funding

Novartis contracted with the IBCSG to support the BIG 1–98 trial. IBCSG had responsibility for the trial design; to collect, analyze, and interpret the data; and report the results. This report presents translational research based on collected tumor material (collection partially funded by Novartis), nucleic acid extraction, and gene expression assay (funded by Susan G. Komen for the Cure Promise Grant [KG080081 to GV, MMR]). SB is supported by a Susan G. Komen Scholar award (SAC 110004).

## Notes

Affiliations of authors: University of Notre Dame, Notre Dame, IN (SB); Harper Cancer Research Institute, Notre Dame, IN (SB); IBCSG Statistical Center Department of Biostatistics and Computational Biology, Dana Farber Cancer Institute, Boston, MA (KPG, MMR); Harvard Medical School, Boston, MA (MMR); Harvard T.H. Chan School of Public Health, Boston, MA (KPG); Department of Pathology and Laboratory Medicine, Indiana University School of Medicine, Indianapolis, IN (YGP, SSB); Avera Cancer Institute, Department of Molecular and Experimental Medicine, Sioux Falls, SD (SW, BLJ); International Breast Cancer Study Group Coordinating Center Pathology Office, Bern, Switzerland (RK); Breast Center, Kantonsspital, St. Gallen, Switzerland (BT); Swiss Group for Clinical Cancer Research SAKK, Berne, Switzerland (BT); Indiana University Melvin and Bren Simon Cancer Center, Indianapolis, IN (SSB); University of Milan, IEO European Institute of Oncology IRCCS, Milan, Italy (GV).

All authors read the manuscript before submission. SAB, YGP, and SSB are holders of the NotreDame/Indiana University patent for the gene signature.

We are indebted to the women, physicians, nurses, and data managers who participated in the BIG 1–98 clinical trial; to the many pathologists who submitted tumor blocks; to the BIG 1–98 Collaborative Group and BIG 1–98 Steering Committee; to Novartis for supporting the execution of the clinical trial and the collection of tumor blocks; and to the IBCSG for the design and coordination of the trial and the IBCSG Central Pathology Office for tumor block collection and processing.

## Supplementary Material

pkz051_Supplementary_Data
