# Supplementary material for: Independent Validation of EarlyR Gene Signature in BIG 1-98: A Randomized, Double-Blind, Phase III Trial Comparing Letrozole and Tamoxifen as Adjuvant Endocrine Therapy for Postmenopausal Women With Hormone Receptor–Positive, Early Breast Cancer
Source: JNCI Cancer Spectr. 2019 Aug 16;3(4):pkz051. doi: 10.1093/jncics/pkz051 (PMC7049990; doi:10.1093/jncics/pkz051)

Supplementary Methods

Computation of EarlyR score from gene expression values

For ease of reference, the computation of EarlyR, detailed in Buechler et al[1], is summarized here. The principal components of the computation of EarlyR score, described below, are (1) the transformation of the expression values of a gene to a so-called *risk score,* and (2) the calculation of a score (0-100) from the gene risk scores of the 5 panel genes.

Gene risk score. This concept was derived from that of a multistate gene[1,2]. A gene is considered multistate if the distribution of its expression values across a population is sufficiently bimodal, which is formalized with the statistical concept of a mixture model. In that earlier work, a multistate gene was used in a prognostic model by replacing the continuous vector of expression values for the gene by a binary variable representing the two components. By convention, the component enriched with poor prognosis cases was given the value 1 and the other component the value 0. Following the initial division into high and low components by a mixture model, the binary variable representing a gene can be computed by specifying a threshold dividing the components.

One weakness of this approach is that samples near the defining threshold of a multistate gene have some risk of misclassification due to inherent uncertainty about the value of the threshold. In the computation of EarlyR, the binary variable of a multistate gene is replaced by the probability that a sample is correctly classified, measured as follows. The Gaussian mixture model used to define the two components of the multistate gene also computes, for each sample, a probability (0-1) that the sample is in the component labeled 1. We call this probability the gene's *risk score.* Intuitively, the risk score is the probability that a sample is assessed to have high risk of recurrence by this gene. The contribution of each panel gene to the EarlyR score is through its risk score.

Computation of the EarlyR score. Interpreting the risk score of a panel gene as the probability that a sample is in the gene's high-risk component, the EarlyR score measures the probability that the sample is in the high-risk state of at least two genes, as detailed in Buechler et al[1]. For simplicity, the EarlyR score is scaled to the range 0 to 100 by multiplying the original probability by 100.

Discovery of the EarlyR panel. EarlyR was discovered using a training set of ER+, LN- samples from publicly available GEO datasets GSE3494[3]and GSE7390[4], in which gene expression was measured with Affymetrix microarrays (hgu133a)[2,5].

Initial Validation of the *EarlyR* score assay

Following initial identification of the panel genes and algorithm for computing EarlyR score, its prognostic significance has been assessed in an Affymetrix-based dataset consisting of ER+ samples from publicly available datasets on GEO (GSE12093[6], GSE6532[7] (Oxford cohort), GSE2034[8], GSE11121[9], GSE17705[10]), independent from the training data, and in the ER+ METABRIC[11] dataset[5]. In all of these datasets, EarlyR continuous score and risk stratification were significantly prognostic of distant metastasis-free interval up to 8 years post diagnosis ($P<$ 0.00001). In addition, in the ER+ METABRIC cohort, both the EarlyR continuous score and risk stratification are significantly prognostic of 8-year breast cancer free interval ($P < 10^{-7}$). The assay has also been optimized for formalin-fixed paraffin embedded sections.

References for Supplementary Methods

1. Buechler, S., Gokmen-Polar, Y. & Badve, S. EarlyR - A Robust Gene Expression Signature for Predicting Outcomes of ER+ Breast Cancer. *Clin. Breast Cancer* (**in press**)
2. Buechler, S. A. Low expression of a few genes indicates good prognosis in estrogen receptor positive breast cancer. *BMC Cancer* **9,** 243 (2009).
3. Miller, L. D. *et al.* An expression signature for p53 status in human breast cancer predicts mutation status, transcriptional effects, and patient survival. *Proc Natl Acad Sci USA* **102,** 13550–13555 (2005).
4. Desmedt, C. *et al.* Strong time dependence of the 76-gene prognostic signature for node-negative breast cancer patients in the TRANSBIG multicenter independent validation series. *Clin Cancer Res* **13,** 3207–3214 (2007).
5. Buechler, S., Gökmen Polar, Y. & Badve, S. INDUCT: A risk score to predict relapse in estrogen-receptor–positive breast cancer. *Journal of Clinical Oncology* **32,** (2014).
6. Zhang, Y. *et al.* The 76-gene signature defines high-risk patients that benefit from adjuvant tamoxifen therapy. *Breast Cancer Res Treat* **116,** 303–309 (2009).
7. Loi, S. *et al.* Definition of clinically distinct molecular subtypes in estrogen receptor-positive breast carcinomas through genomic grade. *J Clin Oncol* **25,** 1239–1246 (2007).
8. Wang, Y. *et al.* Gene-expression profiles to predict distant metastasis of lymph-node-negative primary breast cancer. *Lancet* **365,** 671–679 (2005).
9. Schmidt, M. *et al.* The humoral immune system has a key prognostic impact in node-negative breast cancer. *Cancer Res* **68,** 5405–5413 (2008).
10. Symmans, W. F. *et al.* Genomic Index of Sensitivity to Endocrine Therapy for Breast Cancer. *J Clin Oncol* **28,** 4111–4119 (2010).
11. METABRIC Group *et al.* The genomic and transcriptomic architecture of 2,000 breast tumours reveals novel subgroups. *Nature* **105,** 1–7 (2012).

Supplementary Tables

Table S1. Characteristics of BIG 1-98 ITT population and EarlyR cohort which was sampled using a case-cohort design (Unweighted distribution)

Characteristics	BIG 1-98 ITT (N=8010)	EarlyR (N=1174)
Age, median (IQR), yrs	61 (56,67)	61 (56,68)
Prior chemotherapy (%)	25	34
Tumor size >2 cm (%)	37	40
Tumor grade, 2 or 3 (%)	79	85
Lymph node positive (%)	42	53
HER2 negative (%)	93	94
Ki-67 LI % immunostained cells, median (IQR)	12 (7,18)	13 (7,21)
Breast cancer event (%)	15	18

Supplementary Figures

Figure S1. Weighted Kaplan-Meier(KM) estimates of DRFI for the ER+ samples in the monotherapy arms of tamoxifen (Tam) vs. letrozole (Let) in (a) EarlyR –Low, (b) EarlyR-High, and (c) EarlyR-Int risk strata.

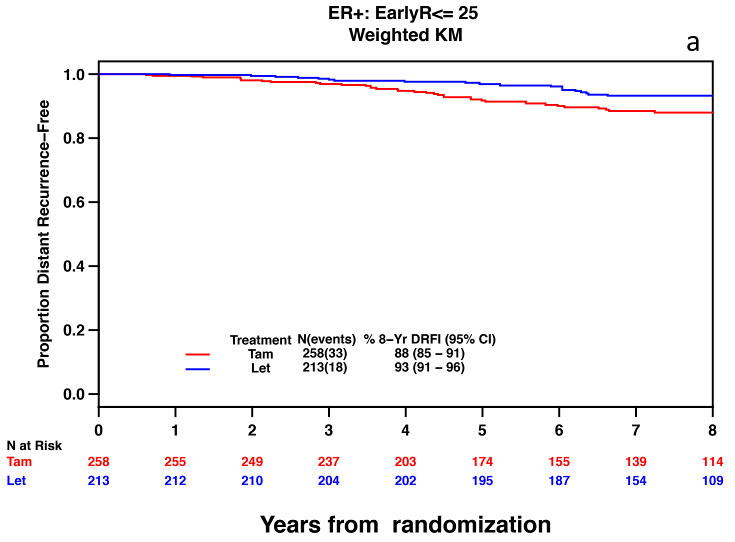

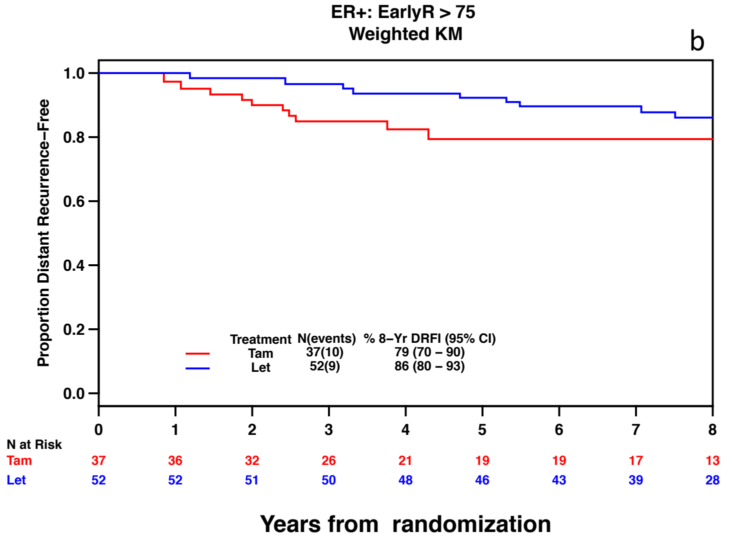

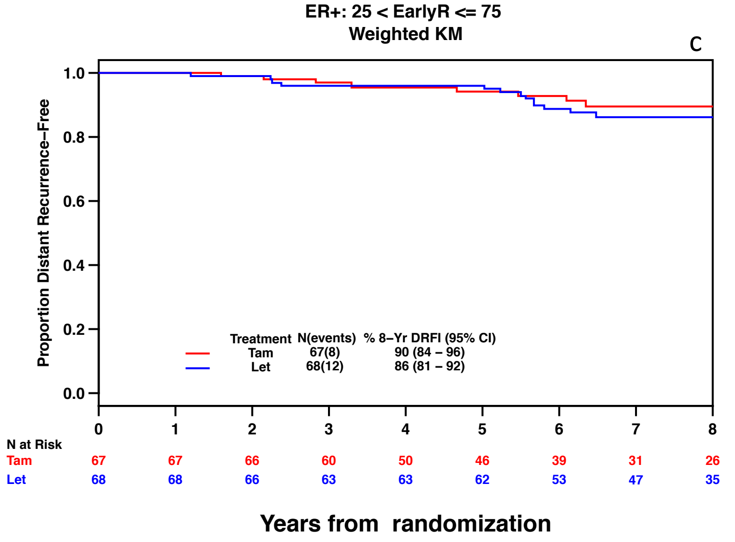

Figure S2. Weighted Kaplan-Meier(KM) estimates of (a) DRFI and (b) BCFI according to EarlyR risk strata in ER+ LN- HER2- patients.

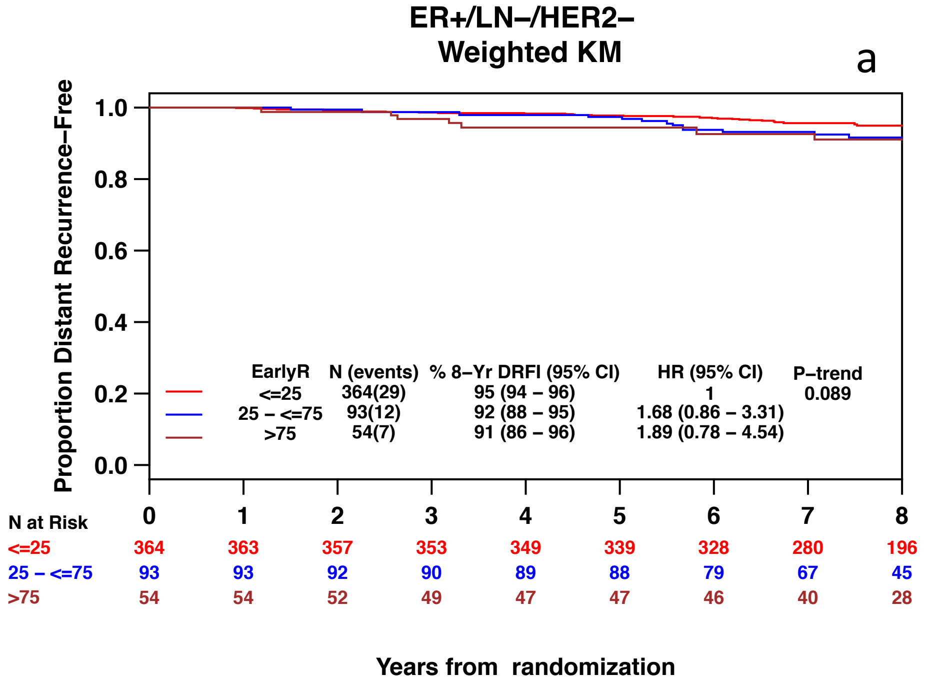

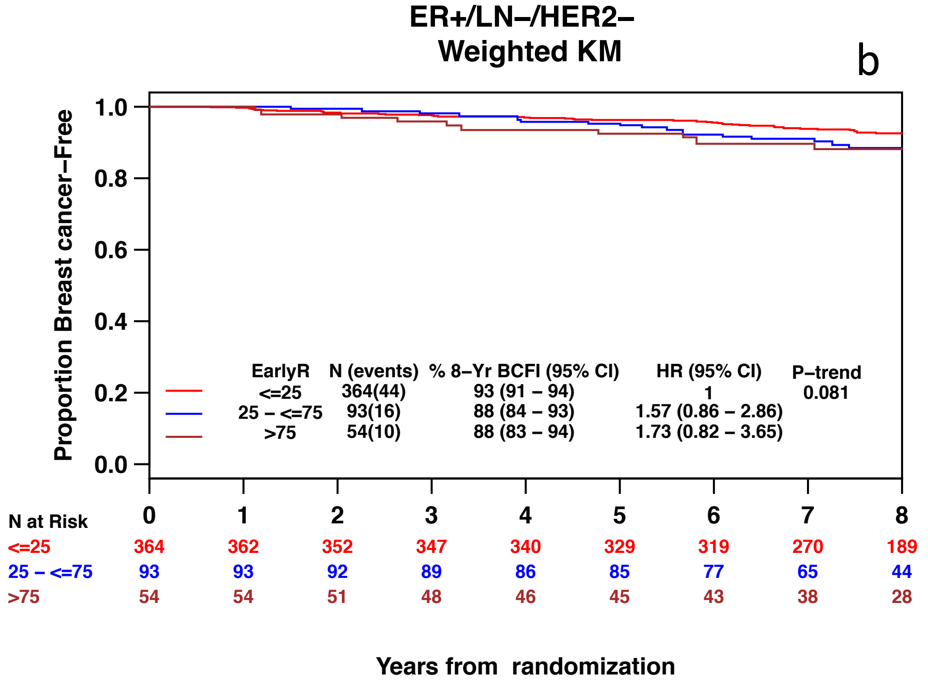

Supplement: pkz051_Supplementary_Data [file pkz051_supplementary_data.pdf]
